# Teratogenic Effect of High Dose of *Syzygium guineense* (Myrtaceae) Leaves on Wistar Albino Rat Embryos and Fetuses

**DOI:** 10.1155/2021/6677395

**Published:** 2021-03-24

**Authors:** Melese Abebe, Kaleab Asres, Yonas Bekuretsion, Samuel Woldkidan, Eyob Debebe, Girma Seyoum

**Affiliations:** ^1^Department of Anatomy, College of Health Sciences, Addis Ababa University, Addis Ababa, Ethiopia; ^2^Department Pharmaceutical Chemistry and Pharmacognosy, College of Health Sciences, Addis Ababa University, Addis Ababa, Ethiopia; ^3^Department of Pathology, College of Health Sciences, Addis Ababa University, Addis Ababa, Ethiopia; ^4^Traditional and Modern Medicine Research Directorate, Ethiopian Public Health Institute, Addis Ababa, Ethiopia

## Abstract

*Syzygium guineense* is an important medicinal plant effective against hypertension, diabetes mellitus, and cancer but with no evidence of its teratogenicity. This study was planned to investigate the teratogenic potential of *S. guineense* leaves on rat embryos and fetuses. Five groups of Wistar albino rats, each consisting of ten pregnant rats, were used as experimental animals. Groups I-III rats were treated with 250, 500, and 1000 mg/kg of hydroethanolic extract of *S. guineense* leaves, and groups IV and V were control and ad libitum control, respectively. Rats were treated during day 6–12 of gestation. Embryos and fetuses were retrieved at day 12 and day 20 of gestation, respectively. The embryos were assessed for developmental delays and growth retardation. The fetuses were examined for gross external, skeletal, and visceral anomalies. In 12-day old rat embryos, crown-rump length, number of somites, and morphological scores were significantly reduced by the treatment of 1000 mg/kg of the extract. The external morphological and visceral examinations of rat fetuses did not reveal any detectable structural malformations in the cranial, nasal, oral cavities, and visceral organs. The ossification centers of fetal skull, vertebrae, hyoid, forelimb, and hindlimb bones were not significantly varied across all groups. However, even if not statistically significant, high-dose treated rat fetuses had a reduced number of ossification centers in the sternum, caudal vertebrae, metatarsal, metacarpal, and phalanges. Treatment with the hydroethanolic extract of *S. guineense* leaves produced no significant skeletal and soft tissue malformations. The plant extract did not produce significant teratogenic effects on rat embryos/fetuses up to 500 mg/kg doses but retarded the growth of embryos at high dose (1000 mg/kg) as evidenced by decreased crown-rump length, number of somites, and morphological scores. Therefore, it is not advisable to take large doses of the plant during pregnancy.

## 1. Introduction

In different countries of Africa, nearly 80% of the people depend largely on herbal therapies for basic healthcare [1]. Behind the accessibility and importance of herbal therapies, especially in Africa, the commonest snags in the use of herbal medicine are their insufficient quality control and safety [2]. Thus, herbal remedies should be standardized to meet the requirements of adequate safety and efficacy [3].

*Syzygium guineense* is a member of the family Myrtaceae. It is an evergreen water-loving dicotyledon tree. In Africa, the plant is widely distributed in Nigeria, Senegal, Eritrea, Ethiopia, Somalia, Zaire, Rwanda, Zambia, Malawi, Zimbabwe, Namibia, Uganda, Swaziland, Cameroon, and South Africa [4]. Traditionally*, S. guineense* is used as a febrifuge and anti-abortifacient medication [5]. It is also used for treating menstrual cycle disorder [6], malnutrition, nasopharyngeal infections, pain, pulmonary disorders [7], constipation, diarrhea, dysentery [8], arthritis, rheumatism, venereal diseases, malaria [9], asthma, wound [10, 11], cancer, infertility [12, 13], sleep disorder, and anemia [5]. Scientifically, the different parts of *S. guineense* (leaves, root, bark, stem, and twigs) have shown proven efficacy against pathogens (bacteria and fungi) [14–18], hypertension [19], and diabetes mellitus [20]. It is also effective against snake venom, pain, inflammation [21], breast and colon cancers [22], and scavenge free radicals [23]. The phytochemical constituents of *S. guineense* are identified by various investigators. The leaf of *S. guineense* contains alkaloids, terpenoids, anthraquinones, flavonoids, tannins, saponins, glycosides, triterpenes, sugars, proteins, lipids, polyphenols, and essential oils [15, 17, 21].

Following cardiovascular and hepatic toxicities, teratogenicity is the third rank concern of drug preparations [3]. Pharmacological screening of any drug for its teratogenic potential has become mandatory before use in humans [24]. Thus, teratogenicity study is one of the most pompous investigations to be performed on herbs. Although *S. guineense* leaf extract is widely used in Ethiopian traditional medicine, its safety upon prenatal exposure is not confirmed yet. Based on this consideration, the current study was conducted to investigate the teratogenic effects of *S. guineense* leaves on rat embryos and fetuses.

## 2. Materials and Methods

### 2.1. Plant Collection and Extraction

The plant was collected from the field in the outskirt of Woliso town, Oromia regional state, 113 km west of Addis Ababa, the capital city of Ethiopia. Plant identification and authentication were done by the National Herbarium, Department of Plant Biology and Biodiversity Management, Addis Ababa University, Ethiopia, where a voucher specimen (MS 001) was deposited. The leaves of *S. guineense* were cleaned, shade-dried, manually broken into pieces, and coarsely powdered by an electric mill. The powder was mixed with 70% ethanol in 1 : 10 powder to solvent ratio and rotated frequently for 24 hours by an orbital shaker at 130 g. Hydroalcoholic extract was used to get maximum amount of extract of both polar and non-polar components of the plant [23]. The mixture was filtered by Whatman paper (No. 1, 18 cm diameter). Then, the filtrate was concentrated with a rotatory evaporator (Büchi Rota Vapor R-205, Switzerland) at 40°C temperature and 175 millibar pressure; then, the concentrate was further dried in a hot water bath at 45°C. The dried extract (solvents were completely removed) was packed in a sealed glass and kept in a refrigerator at −4°C till used for the experiment [25].

### 2.2. Experimental Animals

Wistar albino rats, which were healthy, nulliparous, 220–240 g weight, and 10–12 weeks of age, were used as experimental and control animals. The rats were obtained from the Ethiopian Public Health Institute (EPHI) animal breeding unit and housed for the experiment in the animal facility of the Traditional and Modern Medicine Research Directorate (TMMRD) of EPHI. The rats were acclimatized for one week prior to the actual experiment. The rats were maintained in a stainless-steel cage at room temperature (23 ± 3°C) with a relative humidity of 50 ± 10% under a controlled alternating 12-hour light-dark cycle. Throughout the experiment, rats were served with a conventional laboratory diet (composition: 75% carbohydrate, 16% protein, 55% fat, 3.6% calcium, and 0.4% phosphorus) and an unlimited supply of drinking water.

For mating, each female rat was placed with one randomly selected unrelated male rat with proven fertility. Following an overnight mating, female rats were observed for the presence of a sperm plug. A vaginal smear test was performed to check for the presence of spermatozoa and confirm pregnancy. The date of sperm detection in the vaginal smear was considered as day one of pregnancy.

### 2.3. Experimental Design

The current experiments were conducted in 12-day-old rat embryos and 20-day-old rat fetuses. For each experiment, 50 pregnant rats, with their own identification marked on the tail, were randomly assigned into five groups, each consisting of ten rats, based on the recommendation of Organization for Economic Co-operation and Development (OECD) guideline for developmental toxicity screening test [26]. Pregnant rats were randomized using a computer-based random order generator. The three-treatment groups received the test substance at doses of 250, 500, and 1000 mg/kg body weight and the fourth one (a vehicle control group) received distilled water (1 ml/100 g body weight). The fifth group was ad libitum control (untouched control). The doses of the plant extract were selected based on previous study reports [20]. Also, the median lethal dose (LD50) of the plat is greater than 5000 mg/kg body weight [27]. The treatment periods, for both day-12 and day-20 experiments, were from day 6 to day 12 of gestation. Pregnant rats were daily inspected for any treatment-related adverse effect. Outcome measurement in both experiments was blindly done by the investigator unaware of the treated and control rats.

### 2.4. Day-12 Experiment

At the end of the treatment period (day 12 of gestation), at 12 : 00 hours, the gravid rats were euthanized by intraperitoneal injection of pentobarbital (150 mg/kg of body weight) [28]. The abdomen was opened by laparotomy and the uterine horns were removed. The uterine horns were placed in a glass containing normal saline and incised along the antimesometrial border to reveal the embryos (experimental unit). With the help of dissecting microscope and fine forceps, the surrounding membranes of the embryo were removed to expose the visceral yolk sac. The development of allantois and yolk sac circulation was evaluated. In the yolk sac removed embryos, embryonic development was evaluated based on Brown and Fabro's [29] morphological scoring system of the rat embryo which was adopted for use in an *in vivo* study by Seyoum and Persaud [30]. The development of body systems (circulatory, nervous, and skeletal) and craniofacial development was assessed. In addition, crown-rump-length (CRL) was measured and the number of somites was counted.

### 2.5. Day-20 Experiment

#### 2.5.1. Morphological Evaluation on 20-Day-Old Rat Fetuses

The pregnant rats were treated from day 6–12 of gestation and sacrificed at day 20 of pregnancy following euthanasia by pentobarbital. The abdomen was opened by laparotomy and the uterine horns were removed. By placing the uterine horns in the clean glass, an incision was made along the antimesometrial border of each horn. Thereupon, each fetus (experimental unit) was revealed and separated from the corresponding placenta. All fetuses were inspected head to tail for any gross developmental abnormalities. The examination includes craniofacial developmental anomalies (exencephaly, anencephaly, microphthalmia, and anophthalmia), limb development abnormalities (syndactyly, adactyly, polydactyly), vertebral column malformations (neural tube defect, kyphosis, scoliosis), tail development disorder (missing tail), and external genitalia abnormalities.

Subsequently, 2–3 fetuses from each rat were preserved in 95% ethanol for skeletal development examination. The rest fetuses were fixed in Bouin's solution (aqueous saturated solution of picric acid 75%, formalin 25%, and glacial acetic acid 5%) for visceral examination.

#### 2.5.2. Visceral Examination on 20-Day-Old Rat Fetuses

After an external examination of the fetuses at necropsy, additional soft tissue examination was conducted by serial sectioning. Serial sectioning was performed on the fetuses fixed in Bouin's solution for two weeks. The sectioning procedure was done by a surgical blade, based on the Modified Wilson technique [31]. Craniocaudally, sections were done at 1–2 mm interval under a dissecting microscope (XTL3101, 6x magnification). The first section was made through the jaw and pass posteriorly above the ear. After removing the tongue, the palate was examined for the presence of any cleft. A coronal section on the head and a transverse section on the neck and parts below were done. The following organs were assessed for any visible anomalies: brain (hydrocephalus, dilation of ventricles, microphthalmia/anophthalmia), craniofacial region (nasal septum defect, cleft palate), thoracic region (lungs: lobar defect, heart: septal defect, retroesophageal aortic arch), abdominal region (liver, stomach, and gut anomalies), and pelvic region (kidneys: agenesis, ectopic kidney, and hydronephrosis, gonads: testes and ovarian anomalies).

#### 2.5.3. Skeletal Staining and Evaluation of 20-Day-Old Rat Fetuses

Skeletal staining was done following a modified method of Rigueur and Lyons [32]. Two to three fetuses per litter were selected for skeletal examination. These fetuses were killed by an overdose of pentobarbital and eviscerated by a midline incision made through the anterior abdominal wall. Eviscerated fetuses were fixed in a bottle containing 95% ethanol (EtOH) for five days. To clear the soft tissues, specimens were placed in 1% potassium hydroxide (KOH) solution for 2–3 days, until the bones were clearly visible. To stain the bones, the specimens were transferred into a fresh KOH solution containing a few droplets (0.005%) of alizarin red stain. The bones were stained within 24 hours. Overstaining of the bones was corrected by Mall's solution (79% distilled water, 20% glycerin, and 1% KOH). After staining, the specimens were cleared by an increasing concentration of glycerin (20%, 40%, 60%, and 80%) for about one week in each concentration. Finally, they were kept in 100% glycerin until evaluation. Then, each specimen was examined under a dissecting microscope utilizing bright-field optics and white background.

The degree of ossification of the sternebrae, metacarpal, metatarsal, and sacrococcygeal bones has been reported to be the primary indices of skeletal development in rats [33]. The degree and number of ossification centers in each bone of the fetuses were examined under a dissecting microscope. The skull, hyoid, sternebrae, ribs, vertebrae, and limb bones were evaluated. Assessment of the skeletal development was performed by using a skeletal scoring chart, designed by Nash and Persaud [34]. After investigation, sample photomicrographs were captured with an automated built-in digital dissecting microscope camera (XTL3101, England) under 4x magnification.

### 2.6. Statistical Analysis

One-way analysis of variance (ANOVA) (to check statistically significant difference among all groups) followed by Turkey post hoc test (for significant difference between two groups) was conducted to analyze the data regarding embryonic growth indices. Descriptive statistics followed by a chi-square test was performed to calculate the percentage of malformations and check the difference between groups. Data analysis was done by Statistical Package for Social Sciences (SPSS) version 24 and the results were expressed as mean ± standard deviation of mean and percentage of malformations. *p*-value <0.05 was considered statistically significant.

### 2.7. Ethical Approval

A letter of ethical approval was obtained from the Department of Anatomy Graduate Committee (protocol number 012/19/ANAT) and Institutional Review Board (IRB) of the College of Health Sciences, Addis Ababa University (number AAUMF03-008), in compliance with OECD guideline (2018) [35] for the care and use of laboratory animals. Rats used in this study were kept in the highest standards for the humane use of animals in biomedical research laboratory of EPHI. They were not subjected to any unnecessary painful and terrifying situations. Administration of the test substance was carried out by an expert and maximum effort was applied to prevent them from pathogens. Before rats were sacrificed, to avoid pain and suffering, they were anesthetized with pentobarbital. Finally, unused pups and sacrificed parental rats were disposed of humanely by the laboratory standards of EPHI.

## 3. Results

### 3.1. Growth of the Embryo

The CRL, the number of somites, and morphological scores were considered as embryonic growth indices. The results are presented in Table 1. Generally, the CRL of the embryos was reduced dose-dependently in the treated groups. The CRL of rat embryos treated with 1000 mg/kg body weight of the test plant was significantly lower when compared to the control and ad libitum control groups. It was 5.1 ± 0.4 in the ad libitum control and 4.3 ± 0.4 in the high-dose (1000 mg/kg body weight) treated group. The mean number of somites was significantly lower (*p*-value <0.01) in the high-dose treated group (1000 mg/kg body weight) compared to the ad libitum control group. No significant difference was seen among the other groups.

The average morphological score was calculated based on morphological scoring system developed by Brown and Fabro which was adopted for use in an *in vivo* study by Seyoum and Persaud. The mean morphological score for rats treated with 1000 mg/kg body weight of the plant extract was 44.9 ± 0.5. The score of control and ad libitum control groups were 45.8 ± 0.6 and 46 ± 0.5, respectively. The mean morphological score was significantly lower in pregnant rats treated with 1000 mg/kg of the hydroalcoholic extract of *S. guineense* compared to the control and ad libitum control groups, *p*-value <0.05 (Table 1).

### 3.2. Embryonic Body System Development

**Figure 1:**
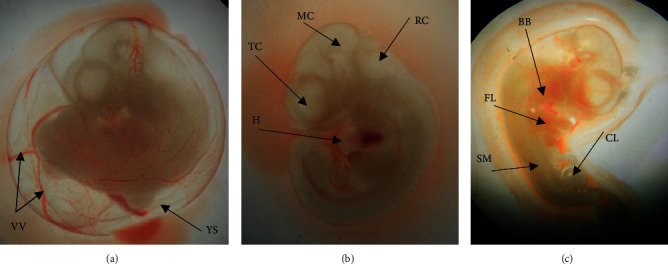
12-day-old rat embryos showing the primordia of different organs. (a) An embryo enclosed inside intact yolk sac (**YS**) with surrounding vitelline vasculatures (**VV**) (b). Embryo without yolk sac, the surrounding blood vessels removed, showing the heart (**H**), telencephalon (**TC**), mesencephalon (**MS**), rhombencephalon (**RC**). (c) Showing branchial bars (**BB**), forelimb bud (**FL**), somites (**SM**), and caudal limb bud (**CL**).

Delays in the development of various systems of the embryo are presented in Tables 2–4. Developmental retardations in embryonic circulatory, nervous, and musculoskeletal systems and craniofacial region were not significantly varied across all groups. However, embryos in the high-dose group showed a higher percentage of retarded development in the olfactory system and somites score compared to the other groups (Table 3).  Figure 1 depicts the developmental status of the primordia of different systems of the embryo.

### 3.3. External and Visceral Morphological Anomalies

**Figure 2:**
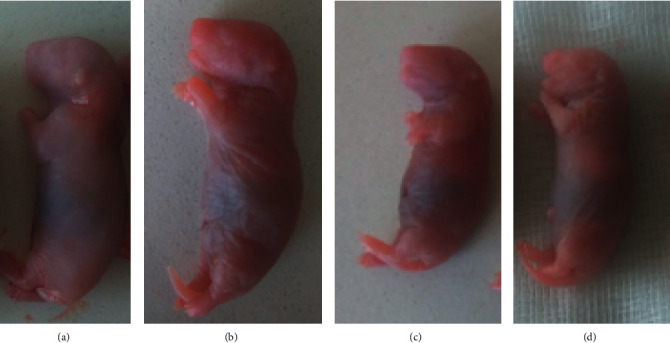
Live rat fetuses from dams in group: (a) control, (b) 250 mg/kg, (c) 500 mg/kg, and (d) 1000 mg/kg.

Explanted fetuses (Figure 2) were freshly examined head to tail for the presence of external malformations. Craniofacial developmental anomalies, limb defect, vertebral column anomalies, missed tail, and external genitalia related abnormalities were inspected. However, our investigation did not reveal any treatment-related external malformations on the near term rat fetuses.

**Figure 3:**
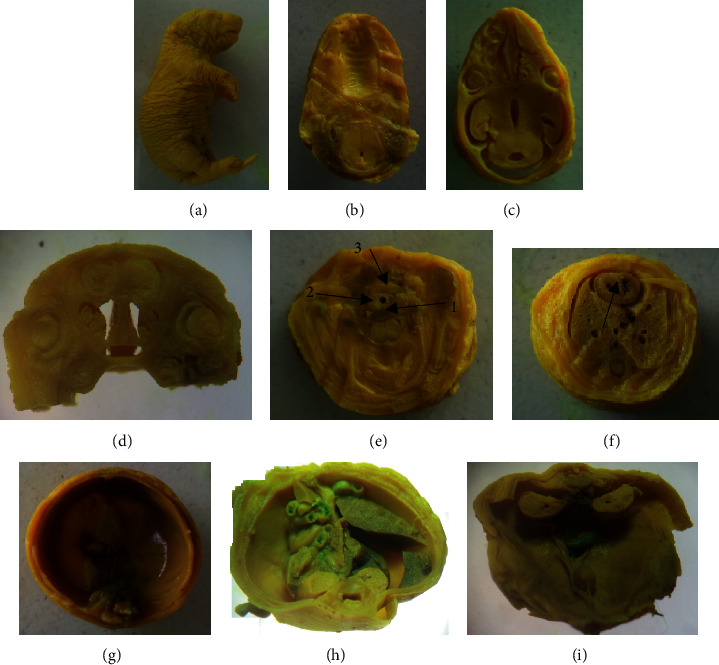
A 20-day-old rat fetus fixed in Bouin's solution for visceral examination (1000 mg/kg). (a) Un-sectioned fetus, (b) normal palate, (c) & (d) transverse section and a coronal section of the brain showing normal ventricle and eyeball, respectively, (e) a section made through the neck showing normal 1-esophagus, 2-trachea, and 3-thyroid, (f) a section through the chest showing normal interventricular septum (arrow), (g) intact diaphragm, (h) & (k) a section made through the abdomen showing normal visceral organs including the kidney.

Fetuses fixed with Bouin's solution were serially sectioned for visceral soft tissue examination. Serial sectioning was made at the level of head, neck, chest, and abdomen. The sections were carefully examined for the existence of any visceral anomalies, under a dissecting microscope. At the head region, cleft palate, hydrocephalus, and eye-related abnormalities were inspected. Additionally, thyroid, thymus, trachea, and cardiac septum associated abnormalities were also checked at the level of the neck and chest. The occurrence of diaphragmatic hernia, agenesis of abdominal viscera, and external genitalia also were investigated. Like the external morphological examination, no visible visceral abnormalities were detected (Figure 3).

### 3.4. Skeletal Malformations

**Figure 4:**
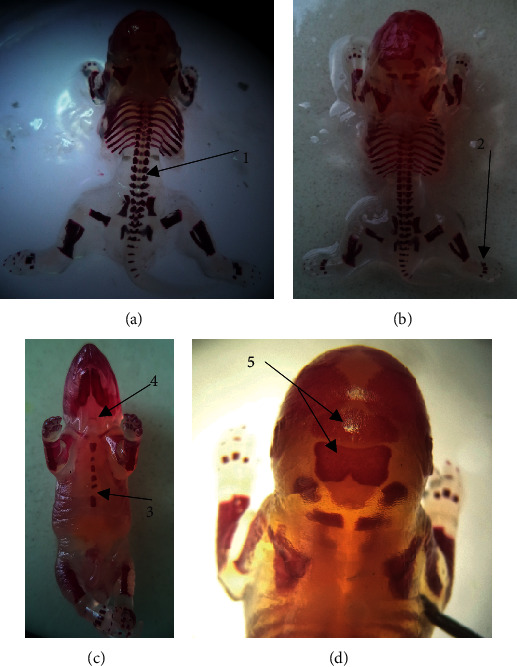
Alizarin red stained rat fetuses showing different ossification centers. 1, vertebrae; 2, metatarsal; 3, sternebra; 4, hyoid; 5, supraoccipital and inter-parietal.

The results of the skeletal evaluation are presented in Tables 5 and 6 and Figure 4. The observation conducted on the skull, thoracic vertebrae, sternum, hyoid, and metatarsals did not reveal statistically significant skeletal malformations between treatment and control groups. The ossification centers of caudal/coccygeal vertebrae, forelimb phalanges, and hindlimb phalanges indicated variation. Fetuses of middle- and high-dose group rats showed decreased number of ossifications in the sternum and caudal vertebrae. However, it was not statistically significant. A total of 56.7% of rat fetuses from mid- and high-dose groups had no proximal hindlimb phalanges. Nevertheless, this variation did not show statistical significance.

## 4. Discussion

Various plant products claimed to have therapeutic significance are obtaining acceptance in the global health market irrespective of their toxicity information [36]. On the other hand, several phytochemical constituents of medicinal plants may be potentially teratogenic. Most of these are commonly used in our day-to-day life [37]. Toxic agents can directly or indirectly affect the embryo/fetus. When the toxic agents cross the placental membrane, they directly damage the developing embryonic/fetal tissue. Indirectly, toxicants that damage the placental tissue and compromise placental function might impede the development of embryos/fetuses [38, 39]. There has been no published study conducted on the teratogenicity of *S. guineense*. The present study investigated the teratogenic potential of the hydroethanolic leaf extract of *S. guineense* in rats. The time of extract administration was day 6–12 of gestation which is the critical period of organogenesis. The assessment of the teratogenic potential of the plant extract was conducted on the skeletal system and soft tissue development of the rat embryos and fetuses.

In the developing embryo, the CRL of the embryo, the number of somites, and morphological scores are important indicators of embryonic growth [40]. Morphological scores have a linear relationship with embryonic age and are used to estimate the growth of the embryo [29]. In general, in the present study, the CRL, the number of somites, and the overall morphological scores of treated rats were decreased in a dose-dependent manner. Rats treated with 1000 mg/kg body weight of the test substance showed a significant reduction in the CRL and average morphological score compared to the control and ad libitum control group. However, rats treated with 1000 mg/kg body weight of the test substance showed significant reduction in the number of somites compared with the ad libitum control group but not with the control group treated with distilled water. Rats in the ad libitum control group were untouched and unrestricted and were not treated as well. Therefore, they were free from stress due to manipulations during intragastric administration of the extract or distilled water. Previous research results indicate that maternal stress can increase the level of stress hormone (cortisol), by which this hormone can pass the placental membrane and affect the development of the embryo, especially the developing brain [41–43]. This may be the reason for the significant difference observed between the high-dose treated group and ad libitum control but not with a control group.

In 12-day-old rat embryos, the development of circulatory system, musculoskeletal system, nervous system, and craniofacial region was assessed under dissecting microscope. Significant developmental delays were not observed across the various groups. However, in the high-dose treated groups, a higher percentage of retarded development was observed in the optic system (8%) and somites score (12%). The leaves of *S. guineense* contain active components like terpenoids, anthraquinones, flavonoids, tannins, saponins, glycosides, triterpenes, and phenols [17]. The current investigation was carried out on a crude extract which makes it difficult to associate the retarded development with a specific group of secondary metabolites. Thus, the above-mentioned phytochemical components of the plant should be isolated and tested for their teratogenic potential.

In 20-day-old rat fetuses, the external morphological examination did not show any visible structural malformations nor did it reveal any treatment-related anomalies in the cranial, nasal, oral cavities, and visceral organs. Congruent with our findings, the plant has been reported to be nontoxic in both acute and sub-acute studies [20, 27]. Therefore, it may be possible to conclude that the plant has no teratogenic effect on the soft tissue of the rat fetuses at the tested doses.

In rodents including rats, many of the bones get ossified in the late fetal period. Therefore, in teratogenicity studies, the extent of bone ossification is an important indicator of fetal maturity [33, 44, 45]. The current study investigated the degree of bone ossification in 20-day-old rat fetuses. The ossification centers of fetal skull, vertebrae, hyoid, forelimb, and hindlimb bones did not vary significantly across the treatment and the control groups. However, treatment with the higher doses (1000 mg/kg and 500 mg/kg body weight) of the plant extract reduced the number of ossification centers of the sternum, caudal vertebrae, metatarsal, metacarpal, and phalanges. Nonetheless, none of these reductions were statistically significant suggesting that the test plant may not have adverse effect on fetal maturity.

## 5. Conclusion

From the results of the present study, it may be possible to conclude that administration of the hydroethanolic extract of *S. guineense* leaves to the pregnant dams does not produce significant skeletal and soft tissue malformations in rat fetuses. The plant extract did not produce significant teratogenic effects on rat embryos/fetuses up to 500 mg/kg doses; however, as high dose (1000 mg/kg) of the plant extract reduced the growth of rat embryo, it is not advisable to take large doses of the plant during pregnancy.

## Figures and Tables

**Table 1:** Embryonic growth following administration of 70% ethanol extract of *Syzygium guineense.*

Group	Variables		
	CRL of the embryo/litter (mm)	Number of somites/litter	Morphological score/litter
Group I (250 mg/kg) *n* = 115	4.7 ± 0.4	28.4 ± 1.3	45.6 ± 0.3
Group II (500 mg/kg) *n* = 113	4.6 ± 0.4	29.1 ± 0.8	45.5 ± 0.3
Group III (1000 mg/kg) *n* = 120	4.3 ± 0.4^*∗∗*^	27.7 ± 0.8^*∗*^	44.9 ± 0.5^*∗∗*^
Group IV (control) *n* = 113	5.08 ± 0.6	28.4 ± 1.2	45.8 ± 0.6
Group V (ad libitum control) *n* = 115	5.1 ± 0.4	29.8 ± 0.9	46 ± 0.6

Results are expressed as mean ± standard deviation of mean. ^*∗*^: mean significantly different from ad libitum control, ^*∗∗*^ significant difference with the control and ad libitum control groups, *p*-value is <0.05. *n*: number of embryos; CRL: crown-rump length.

**Table 2:** Embryonic circulatory system development following administration of 70% ethanol extract of *Syzygium guineense.*

Group	% of retarded development		
	Yolk sac circulation	Allantois	Heart
Group I (250 mg/kg) *n* = 115	0	0	0
Group II (500 mg/kg) *n* = 113	0	0	2
Group III (1000 mg/kg) *n* = 120	2	0	2
Group IV (control) *n* = 113	0	0	0
Group V (ad libitum control) *n* = 115	0	0	0

Results are expressed as a percentage (%) of retarded development (chi-square). *n*: number of embryos.

**Table 3:** Embryonic nervous system and sense organs development following administration of 70% ethanol extract of *Syzygium guineense.*

Group	% of retarded development						
	Caudal neural tube	Hind-brain	Mid-brain	Fore-brain	Otic system	Olfactory system	Optic system
Group I (250 mg/kg) *n* = 115	0	0	0	0	2	2	0
Group II (500 mg/kg) *n* = 113	0	0	0	2	2	4	0
Group III (1000 mg/kg) *n* = 120	0	0	0	2	8	4	0
Group IV (control) *n* = 113	0	0	0	0	0	0	0
Group V (ad libitum control) *n* = 115	0	0	0	0	0	0	0

Results are expressed as a percentage (%) of retarded development (chi-square). *n*: number of embryos.

**Table 4:** Embryonic musculoskeletal system development following administration of 70% ethanol extract of *Syzygium guineense*.

Group	% of retarded development						
	Flexion	Branchial bars	Maxillary process	Mandibular process	Fore-limb	Hind-limb	Somites score
Group I (250 mg/kg) *n* = 115	0	0	0	2	0	2	4
Group II (500 mg/kg) *n* = 113	0	0	2	4	0	4	4
Group III (1000 mg/kg) *n* = 120	0	0	6	4	0	4	12
Group IV (control) *n* = 113	0	0	0	0	0	2	2
Group V (ad libitum control) *n* = 115	0	0	0	0	0	0	2

Results are expressed as a percentage (%) of retarded development (chi-square). *n*: number of embryos.

**Table 5:** Skeletal malformations of 20-day-old rat fetuses following treatment with 70% ethanol extract of *Syzygium guineense*.

Group	% of skeletal malformations				
	Sternum^*∗*^	Hyoid^*∗*^	Ribs	Thoracic vertebrae^*∗∗*^	Caudal vertebrae^*∗∗∗*^
Group I (250 mg/kg) *n* = 30	10	100	100	100	13.3
Group II (500 mg/kg) *n* = 30	23.3	100	100	100	33.3
Group III (1000 mg/kg) *n* = 30	26.7	100	100	100	30
Group IV (control) *n* = 30	16.7	100	100	100	13.3
Group V (ad libitum control) *n* = 30	13.3	100	100	100	16.7

Results are expressed as percentage of skeletal malformations (chi-square). ^*∗*^Sternum with 4 ossification centers and hyoid bone showing signs of ossification. ^*∗∗*^Thoracic vertebrae with 13 ossification centers. ^*∗∗∗*^Caudal vertebrae with 4 ossification centers.

**Table 6:** Skeletal (limb bones) malformations of 20-day-old rat fetuses following treatment with 70% ethanol extract of *Syzygium guineense* extract.

Group	% of skeletal malformations of limb bones			
	Metacarpus^*∗*^+	Metatarsal^*∗*^+	Forelimb^*∗*^! phalanges	Hindlimb^*∗*^! phalanges
Group I (250 mg/kg) *n* = 30	0	0	10	43.3
Group II (500 mg/kg) *n* = 30	13.3	0	16.7	56.7
Group III (1000 mg/kg) *n* = 30	13.3	6.7	20	56.7
Group IV (control) *n* = 30	10	3.3	13.3	20
Group V (ad libitum control) *n* = 30	6.7	0	10	33.3

Results are expressed as a percentage (%) of skeletal malformations (chi-square). ^*∗*^+ Presence of ≤3 metacarpus and metatarsus. ^*∗*^! Absent proximal phalanges.

## Data Availability

All the data are included in the manuscript.

## Abbreviations

ANOVA: Analysis of variance
CRL: Crown-rump length
EPHI: Ethiopian Public Health Institute
EtOH: Ethanol
IRB: Institutional Review Board
KOH: Potassium hydroxide
LD50: Median lethal dose
OECD: Organization for Economic Co-operation and Development
SPSS: Statistical Package for Social Sciences
TMMRD: Traditional and Modern Medicine Research Directorate.
